# Ethanol-based ultrasound-assisted extraction for the sustainable recovery of major flavanone glycosides from mandarin peel^☆^

**DOI:** 10.1016/j.ultsonch.2026.107968

**Published:** 2026-07-21

**Authors:** Hyerim Son, Minji Kim, Soeun Shin, Jeongho Lee, Seunghee Kim, Eunseok Jang, Taek Lee, Hah Young Yoo, Chulhwan Park

**Affiliations:** a Department of Biotechnology, Sangmyung University, 20, Hongjimun 2-Gil, Jongno-Gu, Seoul 03016, the Republic of Korea; b Department of Chemical Engineering, Kwangwoon University, 20 Kwangwoon-Ro, Nowon-Gu, Seoul 01897, the Republic of Korea

**Keywords:** Mandarin peel, Ultrasound-assisted extraction, Flavonoid, Response surface methodology

## Abstract

Mandarin peel (MP), a major citrus-processing by-product, contains flavanone glycosides with antioxidant potential, yet its effective valorization depends on the development of a safe, efficient, and scalable extraction process. In this study, an ethanol-based ultrasound-assisted extraction (UAE) process was developed for the simultaneous recovery of hesperidin, narirutin, and didymin from MP. Response surface methodology based on a central composite design was applied to evaluate the effects of ethanol concentration, temperature, solid loading, and ultrasound amplitude on flavonoid recovery. The developed regression models showed good predictive reliability, with R^2^ values of 0.9321, 0.8996, and 0.9296 for hesperidin, narirutin, and didymin, respectively. The optimal conditions were determined to be 78.22 % ethanol, 68.12 °C, 64.31 g/L solid loading, and 28.16 % amplitude for 10 min. Under these conditions, the experimental recoveries of hesperidin, narirutin, and didymin were 36.15, 17.97, and 2.35 mg/g-biomass, respectively, which agreed with the predicted values by more than 90 %. The resulting MP extract contained 23.41 % total flavonoids and exhibited ABTS radical scavenging activity with an EC_50_ value of 384 µg/mL. No cytotoxicity was observed in L929 cells at concentrations up to 1600 µg/mL, which was more than 4-fold higher than the EC_50_ value. These findings indicate that ethanol-based UAE enables the rapid and simultaneous recovery of antioxidant flavonoids from MP using a generally recognized as safe solvent. This process provides a practical strategy for the sustainable valorization of mandarin peel as a natural antioxidant ingredient.

## Graphical abstract

**Figure:**
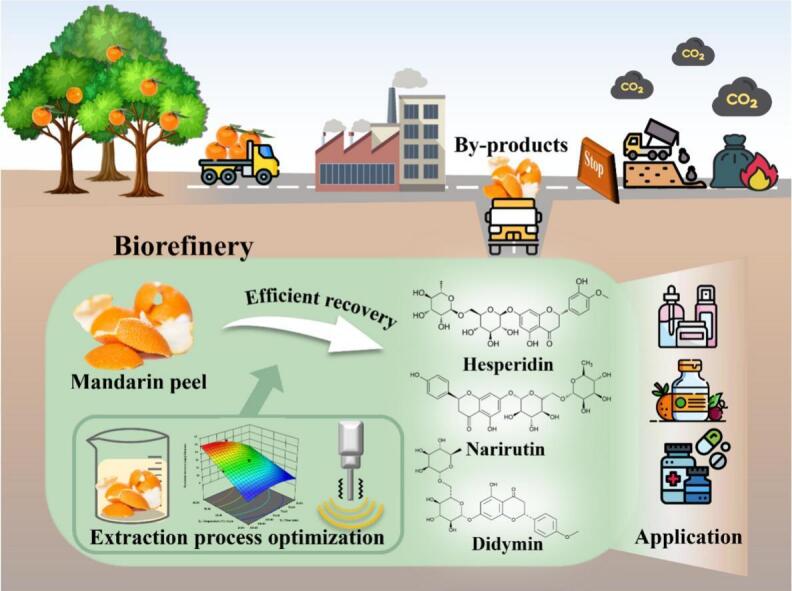

## 1 Introduction

Mandarin is one of the most widely consumed citrus fruits worldwide, with global production estimated at 37.5 million tons [1]. During industrial processing for beverages, flavoring materials, and related products, large amounts of by-products, including peels, pulps, and seeds, are generated, accounting for approximately 50–60 % of the whole fruit weight [2], [3]. These residues are commonly treated by landfill, incineration, or composting, which can cause greenhouse gas emissions and other local environmental burdens [4], [5]. Among these by-products, mandarin peel (MP) is of particular interest because it contains phenolic acids, flavonoids, vitamins, and other bioactive compounds with potential value as natural functional ingredients [6], [7]. Therefore, the valorization of MP represents a practical strategy for reducing citrus-processing waste while producing high-value antioxidant materials.

Flavonoids are plant secondary metabolites that contribute to antioxidant, antibacterial, anti-inflammatory, and other biological activities [8]. MP is known to contain abundant flavanone glycosides, particularly hesperidin, narirutin, and didymin [9], [10], [11]. These compounds are flavanone-7-O-rutinosides, and their radical-scavenging activity is associated with the O-glycosylation at the 7-hydroxyl group and the structural characteristics of their aglycone moieties [12], [13]. The rutinoside moiety, in which rhamnose is linked to glucose through a 1 → 6 bond, also provides a relatively mild sensory profile compared with other flavonoid glycosides, supporting their potential use as antioxidant ingredients in food and cosmetic applications [14]. In addition, hesperidin, narirutin, and didymin have been reported to regulate oxidative stress and inflammatory signaling, suggesting their value as bioactive compounds for functional material development [15], [16], [17], [18], [19], [20], [21], [22].

An efficient and safe extraction process is essential for the practical recovery of flavonoids from MP. In our previous study, the effects of solvent type on hesperidin and narirutin recovery from MP were investigated using maceration, and methanol was found to be highly effective for flavonoid extraction [23]. However, the toxicity of methanol limits its applicability in industries closely related to human use, including food, cosmetics, and pharmaceuticals. Ethanol, a generally recognized as safe (GRAS) solvent, is a more suitable alternative for developing industrially applicable MP extracts. At the same time, conventional solvent extraction often requires long processing times and high solvent consumption. Ultrasound-assisted extraction (UAE) utilizes the cavitation effect caused by the formation, expansion, and collapse of bubbles, which result from the conversion of electrical energy into acoustic energy through mechanical vibrations at ultrasonic frequencies [24]. The cavitation effect disrupts the plant matrix and enhances the diffusion of solutes into the solvent. Through this, UAE has been widely studied as an efficient and green extraction technology that can increase extraction efficiency within a shorter processing time [25]. However, most previous studies on MP have focused on either single flavonoid recovery or extraction systems using less industry-compatible solvents, and the simultaneous recovery of hesperidin, narirutin, and didymin under ethanol-based UAE conditions remains insufficiently optimized.

In this study, an ethanol-based UAE process was developed for the simultaneous recovery of hesperidin, narirutin, and didymin from MP. Response surface methodology (RSM) based on a central composite design was applied to optimize the major extraction variables, including ethanol concentration, temperature, solid loading, and ultrasound amplitude. The effects of these variables and their interactions on the recovery of each target flavonoid were quantitatively evaluated using predictive regression models. The optimized MP extract was further characterized by determining its total flavonoid content, ABTS radical scavenging activity, and cytocompatibility in L929 cells. This study provides a practical sonoprocessing strategy for the sustainable valorization of MP as a source of natural antioxidant ingredients.

## 2 Materials and methods

### 2.1 Materials

The mandarin peel (MP) was purchased from Theyundoo (Uijeongbu-si, Gyeonggi-do, Republic of Korea). The MP was dried at 105 °C for 2 h to completely evaporate the residual moisture in the biomass. It was then ground to a particle size of 425–1,000 μm and stored at −20 °C. The ethanol (EtOH) was obtained from Samchun Chemical (Seoul, Republic of Korea). The standards of hesperidin, narirutin, and didymin for HPLC analysis were obtained from Sigma-Aldrich (St. Louis, MO, USA).

### 2.2 Extraction methods for investigating effect of solvent concentration

To improve the recovery of flavonoids from MP, the effect of solvent concentration was investigated at a solid loading of 100 g/L. Prepared MP was immersed in distilled water (DW) and various concentrations of EtOH (25, 50, 75, 100 %) of 20 mL. The extractions were performed in a water bath for 2 h at 25 °C. Then, the extract was centrifuged at 13,000 rpm for 10 min using a micro-centrifuge (Micro Centrifuge 1730R, Gyrozen, Gimpo, Republic of Korea). The supernatant was used as an analytical sample for quantitative analysis of hesperidin, narirutin, and didymin. The statistical significance between hesperidin, narirutin, and didymin recovery by solvent concentration was analyzed using a one-way analysis of variance (ANOVA) and Tukey's test. The IBM statistics 29.0 (SPSS Inc., USA) was used for statistical analysis and the significance level was set at p-value < 0.05. All experiments were performed in triplicate (n = 3).

### 2.3 Experimental method of ultrasound-assisted extraction

Ultrasound-assisted extraction (UAE) is the method of immersing a sample in a solvent and then offering ultrasound to increase extraction efficiency. Ultrasonication was performed using an ultrasonic processor (Sonics VCX130, Sonics & Materials Inc., Newtown, CT, USA) equipped with a 6-mm-diameter probe (amplitude: 120 μm). The ultrasound is emitted through a transducer, which is submerged about 5 mm below the solvent surface. The UAE was carried out in pulse modes, and the MP was sonicated for 10 min at the 10 s on/10 s off duty cycle. The extraction reaction vials were 50 mL conical tubes, and a water bath was used to provide heat. All extraction experiments were performed at a solvent volume of 20 mL. All experiments were performed in triplicate (n = 3).

### 2.4 Experimental design by response surface methodology

The response surface methodology (RSM) with a central composite design (CCD) was used to optimize the major variables of ultrasound-assisted extraction to enhance the flavonoid recovery from MP, using Design-Expert software version 13 (Stat-Ease Inc., Minneapolis, MN, USA). To optimize the extraction process, *X_1_*: solvent concentration, *X_2_*: temperature, *X_3_*: solid loading, and *X_4_*: amplitude were chosen as major independent variables and separated into five levels (−2, −1, 0, 1, 2) (Table 1). A total of 30 independent experimental combinations were conducted based on five levels of four variables (*X_1_*: 0–80 %, *X_2_*: 30–70 °C, *X_3_*: 50–150 g/L, and *X_4_*: 20–100 %). The effect of variables on flavonoid recovery was investigated by determining the recovery (mg/g-biomass) of hesperidin, narirutin, and didymin recovered from 1 g of MP in each experiment as the response value. The interaction between variables and response values was analyzed using regression analysis, and analysis of variance (ANOVA) was employed to assess the statistical significance of the regression model.

**Table 1:** The major variables and their coded levels in response surface methodology.

Variables	Unit	Symbol	Coded Variable levels				
			−2	−1	0	1	2
Solvent concentration	%	*X_1_*	0	20	40	60	80
Temperature	°C	*X_2_*	30	40	50	60	70
Solid loading	g/L	*X_3_*	50	75	100	125	150
Amplitude	%	*X_4_*	20	40	60	80	100

### 2.5 Analytical methods

#### 2.5.1 Total flavonoid content

The total flavonoid content of mandarin peel extract (MPE) was measured according to the aluminum chloride method as reported by [26], [27]. Briefly, 50 μL of MPE was mixed with 30 μL of 5 %NaNO_2_ (w/w) and reacted at room temperature for 6 min. Then, 50 μL of 10 % AlCl_3_ (w/w) was added to the mixture and a further reaction period of 5 min. After, 300 μL of 1 M NaOH and 1000 μL of DW were added and incubated at room temperature for 15 min. The absorbance was recorded at 380 nm using a spectrophotometer. The flavonoid content was calculated based on hesperidin, the results were presented as mg hesperidin equivalents (HE) per 1 g of MP. All experiments were performed in triplicate (n = 3).

#### 2.5.2 ABTS assay

ABTS radical scavenging activity of MPE was evaluated according to the ABTS assay as described by [28], [29]. ABTS radical solution was prepared by blending 2.45 mM potassium persulfate with 7 mM ABTS solution in a 1:1 ratio and diluted using methanol to an absorbance of 0.8 at a wavelength of 734 nm. Then, 50 μL of MPE was mixed with 950 μL of ABTS radical solution and reacted at room temperature for 30 min. The absorbance was recorded at 734 nm using a spectrophotometer. The ABTS radical scavenging activity (%) was measured based on the following Equation (1):

(1) $$\text{ABTS radical cation scavenging activity}\;\left(\%\right)=\left(\text{1}-{\text{Sample OD}}_{\text{734}}\text{nm}/{\text{Control OD}}_{\text{734}}\text{nm}\right)\times \text{1}00$$

Blank and controls were conducted with the same step using methanol and flavonoids (hesperidin, narirutin, and didymin) standard instead of MPE, respectively. Antioxidant activity results were expressed as EC_50_ values (μg/mL), which indicate the concentration of the extract required to scavenge 50 % of the free radicals. All experiments were performed in triplicate (n = 3).

#### 2.5.3 Cytotoxicity analyses by WST assay

The cytotoxicity of MPE was evaluated using the WST assay with WST-8 from the Cellrix Viability Assay Kit. The mouse fibroblast line L929 obtained from the Korean Cell Line Bank (Seoul, South Korea), was grown in RPMI media containing 10 % fetal bovine serum and 1 % antibiotics. L929 cell suspensions (100 μL; 3000 cells/well) were dispensed into 96-well, and cultured at 37 °C with a 5 % CO^2^ incubator for 24 h. Then, 100, 200, 300, 400, 600, 800, 1200, and 1600 μg/mL of MPE were added into each well and incubated for 24 h. The WST-8 reagent was added 10 μL to the cells, and absorbance was measured at a wavelength of 450 nm using a microplate reader (BioTekGen5, Los Angeles, USA) after 2 h incubation in an incubator. The cell viability was determined based on the following Equation (2):

(2) $$\text{Cell viability}\;\left(\%\right)=A_{1}/A_{0}\times \;\text{1}00$$

where *A_0_* is the absorbance of the control group and *A_1_* is the absorbance of the experimental group with the addition of MPE. All experiments were performed in triplicate (n = 3).

#### 2.5.4 HPLC analysis

The HPLC analysis was conducted using the Hitachi Primaide system (Hitachi, Tokyo, Japan) for detection and measuring the concentration of hesperidin, narirutin, and didymin. The concentration of each compound was determined using standard curves of hesperidin (R^2^ = 0.998), narirutin (R^2^ = 0.992), and didymin (R^2^ = 0.997) constructed by 500, 250, 125, 62.5, and 31.25 ppm. The UV detection wavelength was 280 nm. The autosampler was set at 25 °C and a 5 μL of MPE was injected into INNO Column C18 (5 µm, 4.6 mm × 250 mm) at a flow rate of 0.8 mL/min. Gradient elution was performed to improve the separation efficiency, with acetonitrile as phase A and 0.03 % (v/v) phosphoric acid in DW as phase B. The gradient condition starts with 10 % phase A and up to 20 % until 15 min, 40 % for 28 min, and 75 % until 36 min. Then, returned for 10 % phase A until 38 min and kept for 50 min. Representative HPLC-DAD chromatograms of the standard compounds and MPE are presented in **S1**.

## 3 Results and discussion

### 3.1 Effect of ethanol concentration on flavonoid recovery from MP

**Fig. 1:**
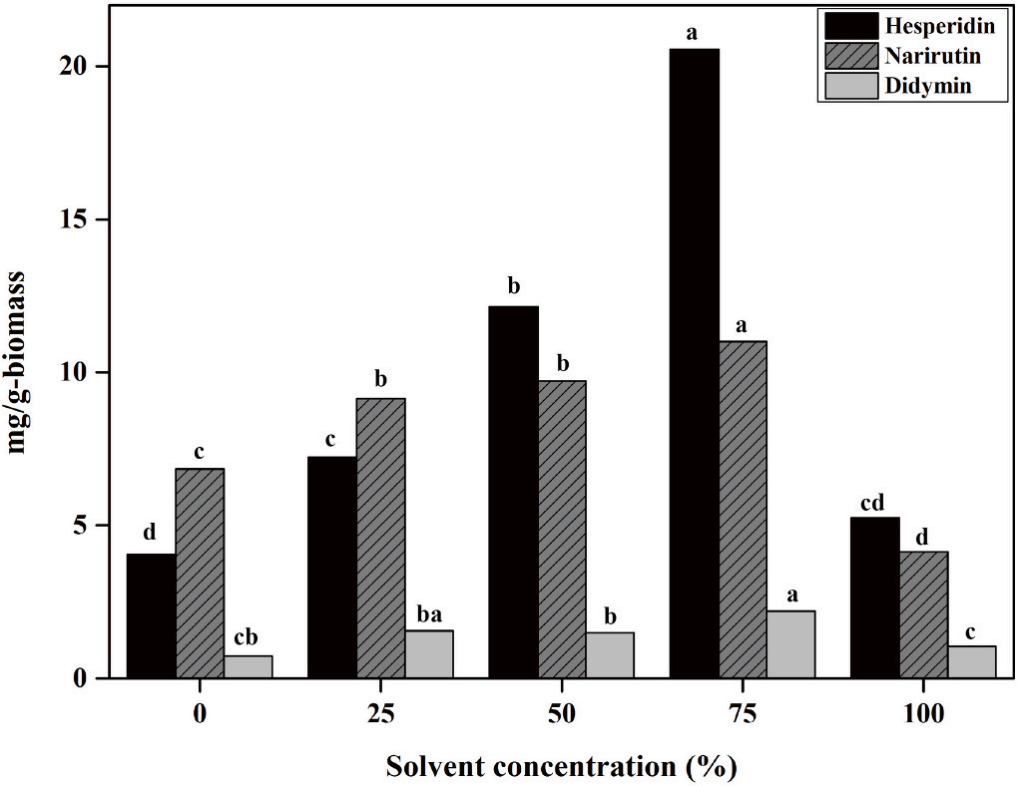
The effect of ethanol concentration on hesperidin, narirutin, and didymin recovery from mandarin peel (Data labeled with different letters indicate statistically significant differences (p < 0.05)).

Binary aqueous organic solvents have been reported to be more effective than single solvents for the extraction of polyphenols, including flavonoids [30], [31]. Therefore, an aqueous ethanol system was applied to improve the recovery of flavonoids from MP and to determine the appropriate solvent concentration range for subsequent RSM optimization. The effects of ethanol concentration on the recovery of hesperidin, narirutin, and didymin from MP were investigated under fixed extraction conditions using a solid loading of 100 g/L. As shown in Fig. 1, the recovery of hesperidin increased from 4.06 to 20.55 mg/g-biomass as the ethanol concentration increased from 0 to 75 %, whereas it decreased to 5.25 mg/g-biomass in 100 % ethanol. Similar trends were observed for narirutin and didymin. The recovery of narirutin was 6.85, 9.14, 9.72, 11.00, and 4.14 mg/g-biomass at ethanol concentrations of 0, 25, 50, 75, and 100 %, respectively. The recovery of didymin was 0.74, 1.56, 1.50, 2.20, and 1.06 mg/g-biomass, respectively.

The highest recovery of all three flavonoids was obtained at 75 % ethanol. This result suggests that an appropriate water-to-ethanol ratio is important for improving flavonoid extraction from MP. The presence of water in the binary solvent may promote swelling of the plant matrix and improve solvent penetration, while ethanol can enhance the solubility of flavanone glycosides. In contrast, 100 % ethanol may limit matrix swelling and intracellular diffusion, resulting in lower flavonoid recovery. A similar trend was reported in a previous study, in which hesperidin recovery from *Citrus reticulata* increased as the ethanol concentration increased from 0 to 75 % [32]. In addition, 80 % ethanol was reported to be effective for recovering total phenolics from mandarin peel and producing extracts with high antioxidant activity [33], [34]. These results indicate that ethanol concentration is a key variable affecting the recovery of hesperidin, narirutin, and didymin from MP. Therefore, ethanol concentration was selected as a major independent variable for UAE process optimization.

### 3.2 Optimization of ultrasound-assisted extraction conditions by response surface methodology

Response surface methodology (RSM) based on a central composite design was used to optimize the UAE process for the simultaneous recovery of hesperidin, narirutin, and didymin from MP. Ethanol concentration (X_1_), temperature (X_2_), solid loading (X_3_), and amplitude (X_4_) were selected as the major process variables because they can affect solvent polarity, mass transfer, and cavitation intensity during UAE. A total of 30 experiments were conducted according to the RSM design, and the experimental and predicted recovery of the three target flavonoids are summarized in Table 2. Multiple regression analysis was performed to describe the relationship between the variables and flavonoid recovery. The regression equations for hesperidin (*Y_H_*), narirutin (*Y_N_*), and didymin (*Y_D_*) recovery are shown in Equations (3), (4), and (5), respectively.

(3) $$Y_{H}=\;\text{13}.\text{66}\;+\;\text{4}.\text{98}X_{1}+\;\text{2}.0\text{8}X_{2}-2.\text{69}X_{3}+0.\text{2254}X_{4}+\text{1}.20X_{1}X_{2}-\text{1}.\text{18}X_{1}X_{3}+0.\text{5947}X_{1}X_{4}-0.\text{6275}X_{2}X_{3}-0.000\text{7}X_{2}X_{4}+0.\text{6659}X_{3}X_{4}-0.\text{2233}X_{1}^{2}-0.0\text{367}X_{2}^{2}+0.\text{98}0\text{7}X_{3}^{2}-0.\text{57}0\text{9}X_{4}^{2}$$

(4) $$Y_{N}=\;\text{11}.\text{97}\;+0.\text{6845}X_{1}+0.\text{7552}X_{2}-0.\text{9485}X_{3}+0.\text{3339}X_{4}+0.0\text{732}X_{1}X_{2}-0.\text{3966}X_{1}X_{3}+0.\text{269}0X_{1}X_{4}-0.\text{2616}X_{2}X_{3}+0.0\text{331}X_{2}X_{4}+0.\text{6292}X_{3}X_{4}-0.\text{2413}X_{1}^{2}-0.0\text{7}00X_{2}^{2}+0.\text{2679}X_{3}^{2}-0.\text{1794}X_{4}^{2}$$

(5) $$Y_{D}=0.\text{7749}\;+0.\text{3595}X_{1}+0.20\text{13}X_{2}-0.00\text{28}X_{3}+0.0\text{5}0\text{6}X_{4}+0.0\text{836}X_{1}X_{2}-0.0\text{841}X_{1}X_{3}+0.0\text{483}X_{1}X_{4}-0.0\text{443}X_{2}X_{3}+0.00\text{17}X_{2}X_{4}+0.0\text{985}X_{3}X_{4}-0.0\text{654}X_{1}^{2}-0.00\text{25}X_{2}^{2}+0.0\text{1}0\text{1}X_{3}^{2}-0.0\text{442}X_{4}^{2}$$

**Table 2:** Experimental design by response surface model and its recovery of hesperidin, narirutin, and didymin from mandarin peel under designed conditions.

Std	Coded factor levels				Response					
					Hesperidin (mg/g-biomass)		Narirutin (mg/g-biomass)		Didymin (mg/g-biomass)	
	*X_1_*	*X_2_*	*X_3_*	*X_4_*	Predicted	Experimental	Predicted	Experimental	Predicted	Experimental
1	–1	–1	–1	–1	9.87	9.13	11.27	11.26	0.17	0.13
2	1	–1	–1	–1	18.59	18.07	12.74	12.44	0.79	0.76
3	–1	1	–1	–1	12.88	9.95	13.09	12.32	0.49	0.26
4	1	1	–1	–1	26.39	28.56	14.86	15.51	1.45	1.68
5	–1	–1	1	–1	6.78	6.03	9.43	9.33	0.22	0.19
6	1	–1	1	–1	10.78	12.06	9.32	9.54	0.51	0.59
7	–1	1	1	–1	7.28	7.45	10.2	10.33	0.37	0.38
8	1	1	1	–1	16.08	15.42	10.39	9.98	0.99	0.87
9	–1	1	–1	1	7.80	8.17	10.07	9.95	−0.03	0.01
10	1	1	–1	1	18.90	19.43	12.62	12.61	0.79	0.83
11	–1	–1	–1	1	10.81	10.24	12.02	11.91	0.30	0.27
12	1	–1	–1	1	26.70	27.16	14.87	14.44	1.45	1.40
13	–1	1	1	1	7.37	5.91	10.75	10.21	0.42	0.25
14	1	1	1	1	13.76	16.39	11.72	11.95	0.90	1.05
15	–1	–1	1	1	7.87	8.10	11.66	11.44	0.57	0.52
16	–2	–1	1	1	19.05	20.50	12.92	13.04	1.39	1.48
17	–2	0	0	0	2.81	5.85	9.63	10.29	−0.21	0.03
18	2	0	0	0	22.72	19.26	12.37	12.12	1.23	1.02
19	0	–2	0	0	9.36	8.89	10.18	10.28	0.36	0.33
20	0	2	0	0	17.66	17.72	13.2	13.5	1.17	1.23
21	0	0	–2	0	22.95	23.77	14.94	15.28	0.82	0.84
22	0	0	2	0	12.21	10.97	11.14	11.2	0.81	0.82
23	0	0	0	−2	10.92	12.12	10.58	10.66	0.50	0.54
24	0	0	0	2	11.82	10.21	11.92	12.25	0.70	0.68
25	0	0	0	0	13.66	13.97	11.97	11.92	0.77	0.79
26	0	0	0	0	13.66	10.53	11.97	10.58	0.77	0.64
27	0	0	0	0	13.66	13.27	11.97	13.05	0.77	0.90
28	0	0	0	0	13.66	14.81	11.97	12.62	0.77	0.82
29	0	0	0	0	13.66	15.53	11.97	12.52	0.77	0.66
30	0	0	0	0	13.66	13.83	11.97	11.12	0.77	0.84

where *Y_H_*, *Y_N_*, and *Y_D_* are hesperidin, narirutin, and didymin recovery (mg/g-biomass), respectively, and *X_1_*, *X_2_*, *X_3_*, and *X_4_* represent ethanol concentration, temperature, solid loading, and amplitude, respectively.

The statistical significance of the developed regression models was evaluated by analysis of variance (ANOVA). The results of ANOVA for the regression models predicting the recovery of hesperidin, narirutin, and didymin from MP are presented in S2. The high F-value (> 1) and low p-value (< 0.05) referred to the statistical significance of the model [35]. The F-values of the hesperidin, narirutin, and didymin recovery models were 14.7, 9.6, and 14.15, respectively, and all models showed p-values below 0.0001. These results indicate that the regression models were statistically significant. The R^2^ values of the models were 0.9321, 0.8996, and 0.9296 for hesperidin, narirutin, and didymin, respectively, indicating high agreement between the predicted and experimental values [36], [37]. The adequate precision (AP) values of the hesperidin, narirutin, and didymin recovery models were 15.5, 11.4, and 15.1, respectively, which were higher than the recommended value of 4 [38], [39]. These results confirm that the developed models had the reliability to predict flavonoid recovery from MP within the designed UAE condition.

The significance of the model term was analyzed to identify the variable affecting each flavonoid recovery. In the hesperidin recovery model, ethanol concentration (*X_1_*), temperature (*X_2_*), solid loading (*X_3_*), *X_1_X_2_*, *X_1_X_3_*, and *X_3_*^2^ showed a significant influence (p-value < 0.05). In the narirutin recovery model, the *X_1_*, *X_2_*, *X_3_*, amplitude (*X_4_*), and their interactions (*X_1_X_3_*, *X_3_X_4_*) were significant. In the didymin recovery model, *X_1_*, *X_2_*, *X_1_X_2_*, *X_1_X_3_*, and *X_3_X_4_* were statistically significant. Among the major variables, ethanol concentration and temperature significantly affected the recovery of all three flavonoids. This result suggests that solvent composition and thermal effects are primary factors for the simultaneous recovery of flavanone glycosides from MP. The four independent variables were confirmed to have a significant effect on flavonoid recovery, and the extraction conditions were optimized using a model equation that considered all variables.

The individual effects of the variables are shown in S3, with the other variables fixed at the center point (level 0). The recovery of hesperidin, narirutin, and didymin improved proportionately with solvent concentration and temperature. This can be explained by the improved solubility of flavanone glycosides in aqueous ethanol and the enhanced diffusion rate at higher temperatures. An increase in temperature may also promote solvent penetration into the plant matrix and accelerate the release of intracellular flavonoids [40], [41]. In contrast, the recovery of hesperidin and narirutin decreased as solid loading increased. This decrease may be attributed to the reduced solvent availability per unit biomass and the lower mass transfer efficiency under high biomass loading conditions. Ultrasound amplitude showed a compound-dependent effect. Narirutin recovery improved proportionately with amplitude, whereas hesperidin and didymin recovery remained relatively constant. This result indicates that stronger cavitation may be effective for releasing narirutin from the MP, while hesperidin and didymin recovery may be governed more strongly by solvent polarity and temperature than by ultrasound intensity. Therefore, excessive ultrasound amplitude may not be necessary for maximizing the simultaneous recovery of all target flavonoids. This finding is important for process design because moderate amplitude conditions can reduce unnecessary ultrasonic intensity while maintaining extraction performance.

**Fig. 2:**
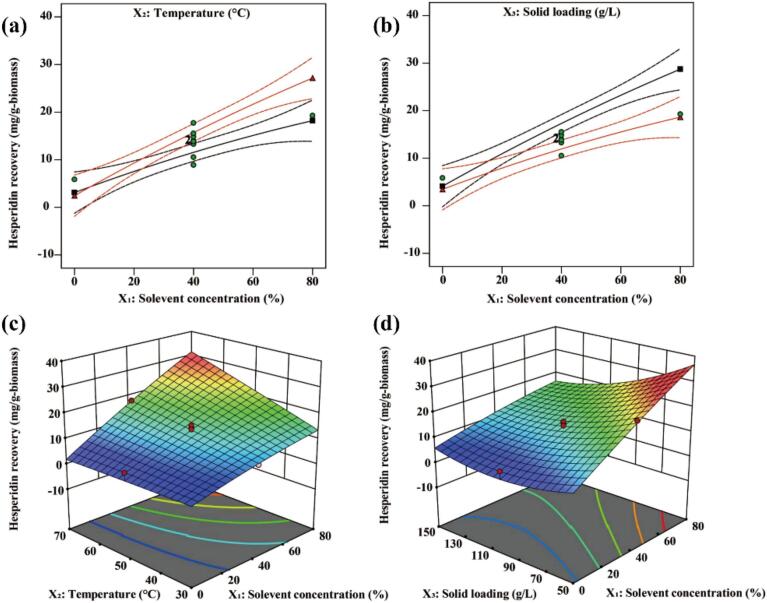
The interactive effect of hesperidin recovery was represented as an interaction plot (a, b) and response surface plot (c, d). The effects of solvent concentration and temperature were represented in the plot (a, c), and solvent concentration and solid loading in the plot (b, d).

The interaction effects between variables were further analyzed using interaction plots and response surface plots. For hesperidin recovery, the interaction between ethanol concentration and temperature was significant, as shown in Fig. 2 (a, c). Hesperidin recovery improves more with high temperature (red line) than with low temperature (black line), according to the increase in ethanol concentration. These results suggest that the combined effects of solvent polarity and thermal diffusion improved hesperidin extraction. The interaction between ethanol concentration and solid loading was also significant (Fig. 2 (b, d)). At lower solid loading, the positive effect of ethanol concentration was more pronounced, indicating that sufficient solvent availability is important for efficient hesperidin recovery.

**Fig. 3:**
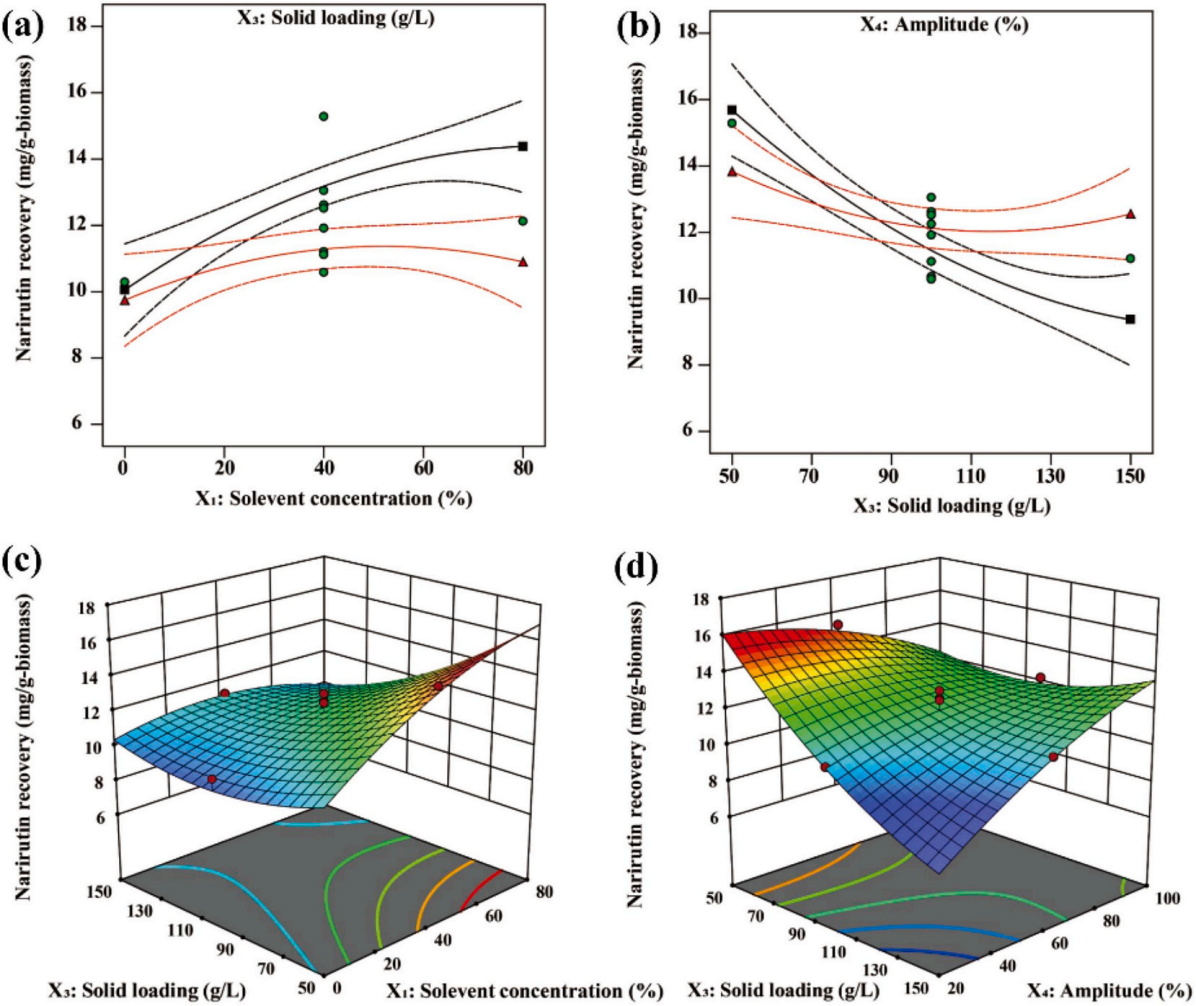
The interactive effect of narirutin recovery was represented as an interaction plot (a, b) and response surface plot (c, d). The effects of solvent concentration and solid loading were represented in the plot (a, c), and solid loading and amplitude in the plot (b, d).

**Fig. 4:**
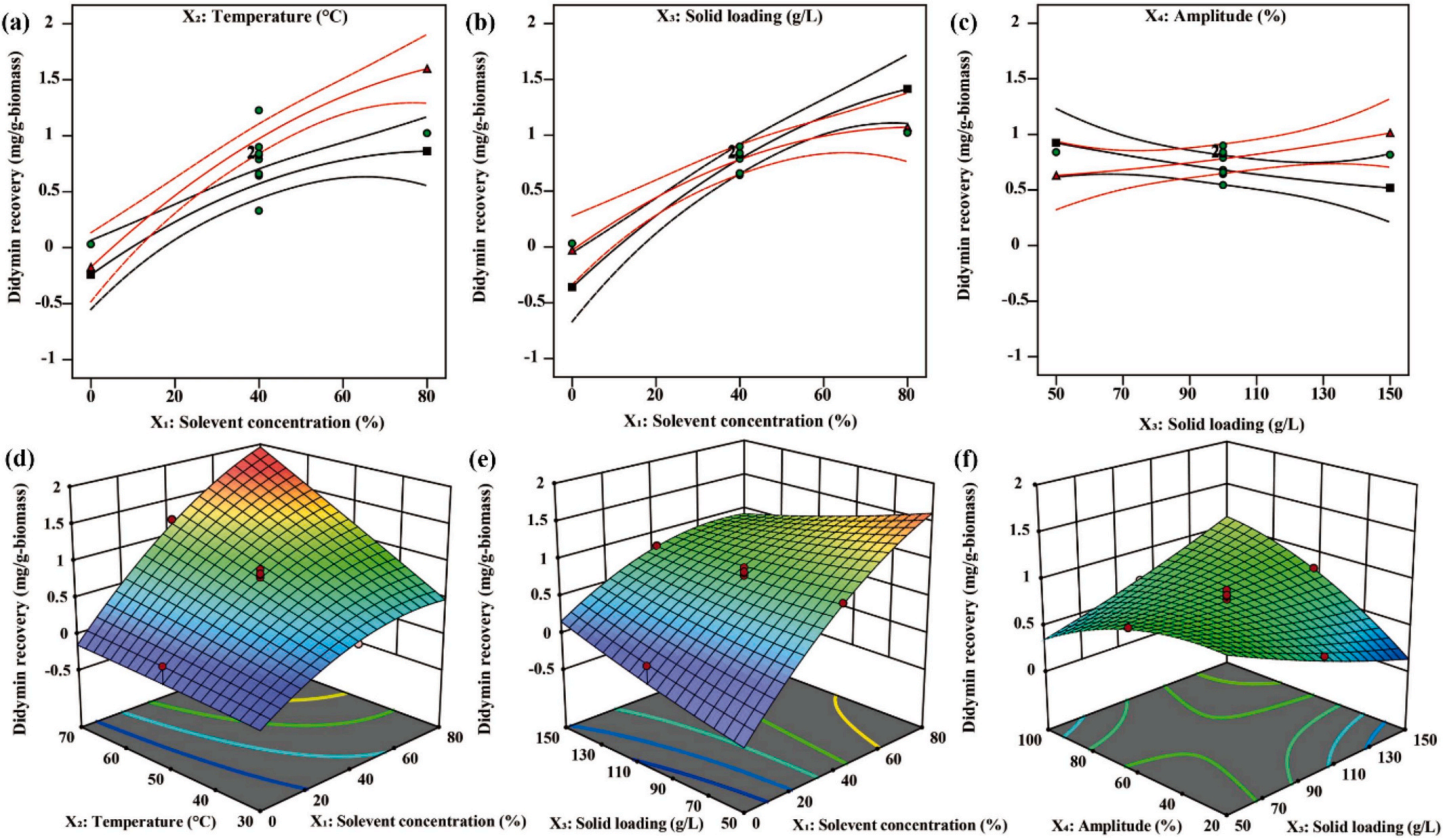
The interactive effect of didymin recovery was represented as an interaction plot (a, b, c) and response surface plot (d, e, f). The effects of solvent concentration and temperature were represented in the plot (a, d), solvent concentration and solid loading in the plot (b, e), and solid loading and amplitude in the plot (c, f).

Fig. 3 illustrates the interaction between the variables that influenced narirutin recovery. The interaction between solvent concentration and solid loading (Fig. 3 (a, c)), and solid loading and amplitude (Fig. 3 (b, d)), is significant for narirutin recovery. The positive effect of solvent concentration decreased as the solid loading increased, which suggests that high biomass loading can limit solvent penetration and reduce extraction efficiency. The high ultrasound amplitude partly compensated for the negative effect of high solid loading, which indicates a cavitation effect enhanced matrix disruption, and microstreaming can improve mass transfer under conditions where solvent access is limited. For didymin recovery, significant interactions were observed between ethanol concentration and temperature, ethanol concentration and solid loading, and solid loading and ultrasound amplitude (Fig. 4). Didymin recovery increased under high ethanol concentration and high temperature, similar to the trend observed for hesperidin (Fig. 4 (a, d)). The interaction between ethanol concentration and solid loading resulted in higher ethanol concentration, and solid loading improved didymin recovery (Fig. 4 (b, e)). The interaction between solid loading and amplitude also shows that ultrasound intensity can influence the release of didymin depending on the solid loadings (Fig. 4 (c, f)).

Overall, the ethanol concentration and temperature were dominant variables for the simultaneous recovery of hesperidin, narirutin, and didymin from MP. Solid loading and amplitude also affected extraction efficiency through their interaction effects. These results indicate that flavonoid recovery from MP is influenced by the combined effects of solubilization by solvent, diffusion due to thermal effects, and mass transfer induced by ultrasound. The predictive model developed by considering each independent variable and the interactions between variables can be used to derive the optimal UAE conditions for the efficient simultaneous extraction of the three target flavonoids.

### 3.3 Statistical optimization and comparison with previous research

Numerical optimization was performed using the developed regression models to determine the UAE conditions that maximize the simultaneous recovery of hesperidin, narirutin, and didymin from MP. The optimal conditions were derived as the variables were set within the designed RSM range, and the recovery of the three target flavonoids was set as the maximum value. The model suggested the range of optimal conditions as follows: solvent concentration of 75–80 %, temperature of 60–70 °C, solid loading of 50–65 g/L, and amplitude of 20–100 %. High ethanol concentration and temperature were found to be the primary drivers of flavonoid recovery, while optimal solid loading remained low to moderate to ensure sufficient solvent availability for mass transfer. Ultrasound amplitude, although involved in interaction effects on recovery of narirutin and didymin, did not required to maximize the overall recovery of the three flavonoids. In the range of optimal conditions, high solids content and low amplitude conditions were chosen to decrease the energy and cost of the extraction process.

The optimal extraction conditions derived through numerical optimization were ethanol concentration 78.22 % (coded levels: 1.91), temperature 68.12 °C (coded levels: 1.81), solid loading 64.31 g/L (coded levels: −1.43), and amplitude 28.16 % (coded levels: −1.59). The predicted recovery of hesperidin, narirutin, and didymin under optimal conditions was 39.57, 18.20, and 2.24 mg/g-biomass, respectively. To validate the predictive accuracy of the developed regression model, the extraction was performed under the optimal conditions, and hesperidin, narirutin, and didymin were found to be 36.15, 17.97, and 2.35 mg/g-biomass, respectively. These results corresponded to more than 90 % of the predicted values, which confirmed that the developed models accurately described flavonoid recovery from MP.

The optimized UAE conditions derived in this study involve high ethanol concentration, temperature, and moderate ultrasound amplitude. The high ethanol concentration and temperature were essential factors in the extraction to enhance the solubility of flavonoids and mass transfer. In the case of amplitude, excessive ultrasonic intensity was not necessary to achieve high flavonoid recovery when solvent concentration and temperature were properly controlled. This result is significant from a process design perspective, as it achieves high extraction efficiency while avoiding unnecessary ultrasound intensity. However, the optimized condition included a relatively high ethanol concentration and temperature. To apply these conditions in a scale-up process, appropriate equipment and safety measures should be used in accordance with industrial safety standards, considering the volatility and flammability of the solvent [42]. In practical manufacturing, the developed predictive models can be used to identify alternative operating conditions with lower temperatures or adjusted ultrasound amplitudes when process safety and energy efficiency are prioritized. Notably, the predictive model developed in this study exhibited high accuracy, suggesting that it can serve as a useful quantitative tool for process design by elucidating the trade-offs among solvent concentration, temperature, solid loading, and ultrasound amplitude.

The extraction performance of the present study was compared with previous studies on the recovery of hesperidin, narirutin, and didymin from MP, as summarized in Table 3. Various extraction methods have been applied for flavonoids recovery, including maceration, stirrer extraction, microwave-assisted extraction, and UAE. In particular, UAE is widely utilized for extraction, as it provides high-yield recovery of bioactive compounds in a short time and preserves their functional properties [43], [44]. The highest hesperidin recovery was 71.60 mg/g-biomass, which was achieved using an extraction solvent as a 1:1 ratio of MeOH and DMSO by microwave-assisted extraction. In another case, 62.12 mg/g-biomass of hesperidin and 24.00 mg/g-biomass of narirutin were recovered with 100 % methanol as the extraction solvent by maceration for 1,440 min. However, the use of MeOH or DMSO is unsuitable for direct application in food, cosmetic, and pharmaceutical industries because of solvent safety concerns. Compared with these previous approaches, the present ethanol-based UAE process provided a practical balance between extraction efficiency, processing time, and industrial compatibility. The recovery of hesperidin, narirutin, and didymin reached 36.15, 17.97, and 2.35 mg/g-biomass, respectively, within only 10 min using ethanol as a generally recognized as safe solvent. Although the hesperidin recovery was lower than that obtained with MeOH or DMSO-based systems, the present process enabled the simultaneous recovery of three major flavanone glycosides under a food-grade solvent. In addition, the short extraction time and moderate ultrasound amplitude support the potential of this process as an efficient and scalable strategy for MP valorization. Most previous studies on flavonoid recovery from MP have focused on the identification of flavonoids in extracts or single-compound recovery. In contrast, this study optimized the simultaneous recovery of hesperidin, narirutin, and didymin using an RSM, and is significant in that it achieved high yields within a short time using GRAS solvents.

**Table 3:** Summary of hesperidin, narirutin, and didymin recovery from mandarin peel.

Method	Conditions				Hesperidin (mg/g-biomass)	Narirutin (mg/g-biomass)	Didymin (mg/g-biomass)	Ref.
	Solvent (*v/v*)	Temp. (°C)	Time (min)	Solid loading (g/L)				
Mac	100 % EtOH	60	120	50	2.04	1.35	0.05	[3]
Mac	80 % EtOH	−	12	42	17.53	3.54	−	[45]
Mac	100 % MeOH	50	1,440	100	62.12	24.00		[46]
SE	70 % Aceton	40	120	20	31.42	5.93		[47]
MAE	50 % MeOH in DMSO	24	0.33	100	71.60	16.30	−	[48]
MAE	70 % EtOH	140	7	100	19.40	3.60	−	[49]
UAE	80 % MeOH	40	60	50	1.45	0.56	−	[50]
UAE^a^	80 % MeOH	24	70	500	4.97	−	−	[51]
UAE	80 % Aceton	40	30	50	13.74	−	−	[52]
UAE	80 % EtOH	45	60	50	0.09	−	−	[31]
UAE	80 % EtOH	41	15	33	1.34	−	−	[33]
UAE^b^	70 % MeOH	150	110	20	15.58	2.34	0.66	[53]
UAE	78.22 % EtOH	68.12	10	64.31	36.15	17.97	2.35	**This study**

Mac, Maceration; SE, stirrer extraction; MAE, microwave-assisted extraction; UAE, ultrasound-assisted extraction.

a Mandarin peel was pretreated with a microwave at 250 W for 10 min.

b Mandarin peel was pretreated with heat at 150 °C for 10 min.

### 3.4 Antioxidant activity of mandarin peel extracts obtained under optimal condition

The total flavonoid content and antioxidant activity of the mandarin peel extract (MPE) obtained under the optimized UAE conditions were evaluated to assess its potential as a natural antioxidant. The MPE contained 23.41 % total flavonoids, which indicates that the optimized ethanol-based UAE process produced a flavonoid-rich extract from MP. Among the target flavonoids, hesperidin, narirutin, and didymin accounted for 8.02 %, 3.99 %, and 0.52 % of the extract, respectively. These three flavonoids represented 34.24, 17.03, and 2.22 % of the total flavonoid content in MPE, respectively. Hesperidin was the most abundant target flavonoid, which is consistent with previous reports showing that hesperidin is a major flavonoid in MPE (about 34.45 %) [33].

**Fig. 5:**
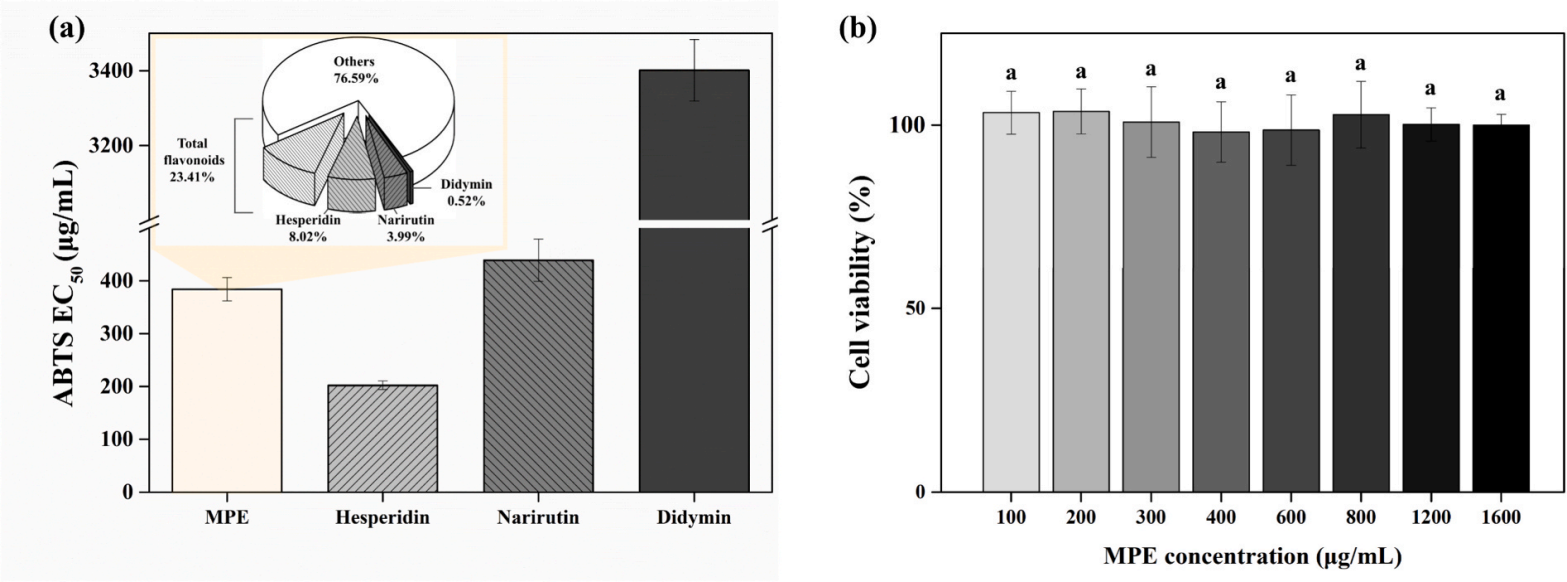
The antioxidant activity of MPE (mandarin peel extract) and reference compounds (hesperidin, narirutin, and didymin) (a), The cytotoxic property of MPE against mouse fibrosarcoma L929 cells (b).

The antioxidant activity of MPE was evaluated using the ABTS assay, and the results were compared with those of hesperidin, narirutin, and didymin as reference compounds. As shown in Fig. 5(a), the ABTS EC_50_ values of MPE, hesperidin, narirutin, and didymin were 384, 202, 439, and 3,401 µg/mL, respectively. The radical scavenging activity of MPE was lower than that of hesperidin but higher than that of narirutin and didymin. Specifically, the ABTS radical scavenging activity of MPE corresponded to 52.60 % of that of hesperidin, while it was 1.14-fold and 8.86-fold higher than those of narirutin and didymin, respectively. Among the reference compounds, hesperidin showed the strongest ABTS radical scavenging activity, followed by narirutin and didymin. This trend may be related to differences in their chemical structures, particularly the presence of hydroxyl and catechol groups in the flavanone backbone, which can influence electron donation and radical stabilization [12]. Although didymin exhibited a relatively high EC_50_ value in the ABTS assay, suggesting weak direct radical scavenging activity, it has been reported to modulate oxidative stress through indirect mechanisms, including the activation of antioxidant enzymes and prevention of reactive oxygen species production [54], [55].

The cytocompatibility of MPE was evaluated in L929 cells using the WST assay to confirm whether the optimized extract could be used at antioxidant-effective concentrations without causing cytotoxic effects. As shown in Fig. 5(b), the cell viability of L929 cells treated with MPE at concentrations of 100, 200, 300, 400, 600, 800, 1200, and 1600 µg/ml was found to be 103.38 %, 103.73 %, 100.80 %, 98.11 %, 98.65 %, 102.81 %, 100.16 %, and 99.99 %, respectively. Cytotoxicity was not observed even at 1600 µg/mL, which corresponds to more than four-fold the ABTS EC_50_ value (384 µg/mL), suggesting that MPE is biocompatible at concentrations sufficient to provide antioxidant effects. Similarly, Diab et al. [56] and Lin et al. [57] reported that ethanol-based MP extracts did not exhibit cytotoxic effects on mouse splenocytes and HaCaT cells at concentrations up to 500 and 10,000 µg/mL, respectively.

In conclusion, the MPE obtained through optimized ethanol-based UAE exhibited high flavonoid content, ABTS radical scavenging activity, and cytocompatibility in L929 cells. These findings support the potential of MPE as a natural antioxidant ingredient produced from MP by a GRAS solvent-based UAE process. To the application of MPE in food and cosmetic products, additional evaluation, including formulation stability, skin irritation, sensitization, and application-specific safety tests, is required to confirm its suitability. In future studies, the stability and safety evaluation of MPE will be conducted to verify its practical applicability as a natural antioxidant ingredient.

## 4 Conclusions

In this study, an ethanol-based ultrasound-assisted extraction process was successfully designed for the simultaneous recovery of hesperidin, narirutin, and didymin from MP. RSM was applied to evaluate the effects of ethanol concentration, temperature, solid loading, and ultrasound amplitude on flavonoid recovery. The developed regression models showed reliable predictive performance, and the experimental recoveries obtained under the optimized conditions agreed with the predicted values by more than 90 %. Under the optimal conditions of 78.22 % ethanol, 68.12 °C, 64.31 g/L solid loading, and 28.16 % amplitude for 10 min, the recovery of hesperidin, narirutin, and didymin reached 36.15, 17.97, and 2.35 mg/g-biomass, respectively. The optimized extract contained 23.41 % total flavonoids and exhibited ABTS radical scavenging activity with an EC_50_ value of 384 µg/mL. In addition, cytotoxicity was not observed in L929 cells at concentrations up to 1600 µg/mL, which is 4-fold higher than the EC_50_ value. This study demonstrated that UAE, using ethanol as a GRAS solvent, provides a sustainable and effective approach to recovering target flavonoids from MP, highlighting the potential of MPE as a valuable natural antioxidant source for food and cosmetic applications.

## CRediT authorship contribution statement

**Hyerim Son:** Writing – original draft, Formal analysis, Conceptualization. **Minji Kim:** Writing – original draft, Methodology. **Soeun Shin:** Methodology, Investigation. **Jeongho Lee:** Methodology, Investigation. **Seunghee Kim:** Software, Investigation. **Eunseok Jang:** Methodology, Formal analysis. **Taek Lee:** Writing – review & editing, Validation, Supervision, Conceptualization. **Hah Young Yoo:** Writing – review & editing, Validation, Supervision, Project administration, Funding acquisition, Conceptualization. **Chulhwan Park:** Writing – review & editing, Validation, Project administration, Funding acquisition.

## Declaration of competing interest

The authors declare that they have no known competing financial interests or personal relationships that could have appeared to influence the work reported in this paper.
